# Supplementary material for: Enhancing quantum annealing performance by a degenerate two-level system
Source: Sci Rep. 2020 Jan 10;10:146. doi: 10.1038/s41598-019-56758-4 (PMC6954224; doi:10.1038/s41598-019-56758-4)
Supplement: Supplementary file 1 — Supplemental Information. [file 41598_2019_56758_MOESM1_ESM.pdf]

Supplemental Material: Enhancing quantum annealing performance by a degenerate two-level system

Shohei Watabe[1,2], Yuya Seki[2], and Shiro Kawabata[2]
[1] Department of Physics, Faculty of Science Division I,
Tokyo University of Science, Shinjuku, Tokyo 162-8601, Japan and
[2] Nanoelectronics Research Institute, National Institute of Advanced Industrial Science and Technology (AIST),
1-1-1 Umezono, Tsukuba, Ibaraki 305-8568, Japan

I. REDUCTION OF QUANTUM WAJNFLASZ–PICK MODEL TO SPIN-1/2 MODEL

We have shown in Eq. (15) in the main text that the quantum Wajnflasz–Pick model with the N spins with $(g_{\rm u}, g_{\rm l}) = (2,1)$ can be reduced into the spin-1/2 model. In the single-spin case, in particular, if we take

$$|\phi_{\rm u}\rangle = \frac{1}{\sqrt{2}}(1,1,0)^{\rm T}, \tag{I.1}$$

$$|\phi_{\rm l}\rangle = (0,0,1)^{\rm T}, \tag{I.2}$$

the Hamiltonian (1) in the main text projected into the Hilbert space spanned by $|\phi_{\rm u,l}\rangle$ is represented by

$$\hat{\mathcal{H}}(s) = \begin{pmatrix} \langle\phi_{\rm u}|\hat{H}(s)|\phi_{\rm u}\rangle & \langle\phi_{\rm u}|\hat{H}(s)|\phi_{\rm l}\rangle \\ \langle\phi_{\rm l}|\hat{H}(s)|\phi_{\rm u}\rangle & \langle\phi_{\rm l}|\hat{H}(s)|\phi_{\rm l}\rangle \end{pmatrix} \tag{I.3}$$

$$= \begin{pmatrix} -h^z s - 2\omega h'(s) & -2\sqrt{2}h'(s) \\ -2\sqrt{2}h'(s) & h^z s \end{pmatrix}. \tag{I.4}$$

It can be clearly found that this representation is exactly the same form as the block matrix shown in Eq. (12) in the main text.

In this Supplementary Information, by generalizing this idea, we show that the quantum annealing problem of the quantum Wajnflasz–Pick model with the N-spins with arbitrary number of degeneracy $g_{\rm u,l}$ can be reduced into the spin-1/2 model. First, consider the Hamiltonian of the quantum Wajnflasz–Pick model

$$\hat{H}(s) = s\hat{H}_z + (1-s)\hat{H}_x, \tag{I.5}$$

where $\hat{H}_x \equiv -\sum_{i=1}^N h_i^x \hat{\tau}_i^x$ with $h_i^x > 0$, and $\hat{H}_z \equiv f(\hat{\tau}_1^z, \dots, \hat{\tau}_N^z)$ can be expanded in the Maclaurin series. Equation (I.5) is a generalization of (1) with (4) and (5) in the main text. We here introduce eigenstates of a single spin $\hat{\tau}_i^z$ at a site i as

$$|u_1\rangle_i, |u_2\rangle_i, \dots, |u_{g_{\rm u}}\rangle_i, \tag{I.6}$$

$$|l_1\rangle_i, |l_2\rangle_i, \dots, |l_{g_{\rm l}}\rangle_i, \tag{I.7}$$

where $|u_k\rangle_i$ for $k = 1, 2, \cdots, g_{\rm u}$ and $|l_k\rangle_i$ for $k = 1, 2, \cdots, g_{\rm l}$ are eigenstates whose eigenvalues of $\hat{\tau}_i^z$ is $+1$ and -1, respectively.

In the following, we first prove a lemma I: if the parameter ω is a real number, the Hamiltonian of the N-spin quantum Wajnflasz–Pick model can be decomposed into two parts

$$\hat{H} = \hat{P}\hat{H}\hat{P} + (\hat{1} - \hat{P})\hat{H}(\hat{1} - \hat{P}), \tag{I.8}$$

where $\hat{P} \equiv \bigotimes_{i=1}^N \hat{P}_i$ with $\hat{P}_i = |\phi_{\rm u}\rangle_i \langle\phi_{\rm u}|_i + |\phi_{\rm l}\rangle_i \langle\phi_{\rm l}|_i$ is a projection operator. A local projection operator $\hat{P}_i$ is spanned by two bases $|\phi_{\rm u,l}\rangle_i$, where

$$|\phi_{\rm u}\rangle_i \equiv \frac{1}{\sqrt{g_{\rm u}}}\sum_{k=1}^{g_{\rm u}} |u_k\rangle_i, \tag{I.9}$$

$$|\phi_{\rm l}\rangle_i \equiv \frac{1}{\sqrt{g_{\rm l}}}\sum_{k=1}^{g_{\rm l}} |l_k\rangle_i. \tag{I.10}$$

It indicates that the Hilbert space of the quantum Wajnflasz–Pick model can be reduced to a subspace spanned by $|\phi_{\rm u,l}\rangle_i$. We also prove a lemma II: if the parameter ω is a real number and the condition $\omega > -1$ holds, the ground state of the initial Hamiltonian $\hat{H}_x$ with $h_i^x > 0$ is an element of the Hilbert space spanned by $|\phi_{\rm u,l}\rangle_i$. According to these two lemmas I and II, in the case where $\omega \in \mathbb{R}$ and $\omega > -1$, the quantum annealing problem in the quantum Wajnflasz–Pick model is represented as a model where the local spin has two states—the spin-1/2 model.

We first consider the lemma I. Since $\hat{P}^2 = \hat{P}$, a necessary and sufficient condition providing (I.8) is given by

$$\left[\hat{P}, \hat{H}\right] = 0. \tag{I.11}$$

The condition (I.11) can be reduced into

$$\left[\hat{P}, \hat{H}\right] = s\left[\hat{P}, \hat{H}_z\right] + (1-s)\left[\hat{P}, \hat{H}_x\right] = 0. \tag{I.12}$$

Here, we will easily prove that $[\hat{P}, \hat{H}_z] = 0$, for the projection operator $\hat{P}$ is composed of bases that diagonalize $\hat{H}_z$. Indeed, since $\hat{H}_z$ is composed of $\hat{\tau}_i^z$, what we need to show is

$$[\hat{P}_i, \hat{\tau}_i^z] = 0. \tag{I.13}$$

A spectral representation of the single-spin operator $\hat{\tau}_i^z$ can be represented by eigenstates of $\hat{\tau}_i^z$, given in the form

$$\hat{\tau}_i^z = \sum_{k=1}^{g_{\rm u}} |u_k\rangle_i \langle u_k|_i - \sum_{k=1}^{g_{\rm l}} |l_k\rangle_i \langle l_k|_i. \tag{I.14}$$

The projection operator $\hat{P}_i$ is also composed of eigenstates of $\hat{\tau}_i^z$. We can thus immediately conclude that (I.13) holds, which provides $[\hat{P}, \hat{H}_z] = 0$. The result (I.13) can also provide the following representation

$$\hat{\tau}_i^z = \hat{P}_i \hat{\tau}_i^z \hat{P}_i + (\hat{1}_i - \hat{P}_i)\hat{\tau}_i^z(\hat{1}_i - \hat{P}_i). \tag{I.15}$$

The remain we need to prove is

$$\left[\hat{P}, \hat{H}_x\right] = -\sum_{j=1}^{N} h_j^x \left[\bigotimes_{i=1}^{N} \hat{P}_i, \hat{\tau}_j^x\right] = 0. \tag{I.16}$$

The necessary and sufficient condition for (I.16) for arbitrary h_j^x is

$$[\hat{P}_i, \hat{\tau}_i^x] = 0, \tag{I.17}$$

because we can expand a term in (I.16) in the following way

$$\left[\bigotimes_{i=1}^{N} \hat{P}_i, \hat{\tau}_j^x\right] = \left(\bigotimes_{i=1}^{j-1} \hat{P}_i\right) \left[\hat{P}_j, \hat{\tau}_j^x\right] \left(\bigotimes_{i=j+1}^{N} \hat{P}_i\right). \tag{I.18}$$

In order to show (I.17), it is convenient to introduce the spectral representation of the single-spin operator $\hat{\tau}_i^x$, given by

$$\hat{\tau}_i^x = \frac{1}{c}(\omega \hat{A}_i + \hat{B}_i + \text{H.c.}), \tag{I.19}$$

where

$$\hat{A}_i \equiv \sum_{\substack{k,k'=1 \\ k>k'}}^{g_\mathrm{u}} \left|u_{k'}\right\rangle_i \left\langle u_k\right|_i + \sum_{\substack{k,k'=1 \\ k>k'}}^{g_\mathrm{l}} \left|l_{k'}\right\rangle_i \left\langle l_k\right|_i, \tag{I.20}$$

$$\hat{B}_i \equiv \sum_{k=1}^{g_\mathrm{u}} \sum_{k'=1}^{g_\mathrm{l}} \left|u_k\right\rangle_i \left\langle l_{k'}\right|_i. \tag{I.21}$$

Here, a constant c is the normalization factor such that the spectral norm of $\hat{\tau}_i^x$ is to be unity. We can also represent $\hat{\tau}_i^x$ as

$$\hat{\tau}_i^x = \frac{1}{c}\left[\frac{1}{2}\Re\omega\left(\hat{A}_i^\mathrm{u} + \hat{A}_i^\mathrm{l}\right) + i\Im\omega \hat{A}_i + \hat{B}_i' + \text{H.c.}\right] \tag{I.22}$$

where

$$\hat{A}_i^\mathrm{u} \equiv g_\mathrm{u} \left|\phi_\mathrm{u}\right\rangle_i \left\langle \phi_\mathrm{u}\right|_i - \sum_{k=1}^{g_\mathrm{u}} \left|u_k\right\rangle_i \left\langle u_k\right|_i, \tag{I.23}$$

$$\hat{A}_i^\mathrm{l} \equiv g_\mathrm{l} \left|\phi_\mathrm{l}\right\rangle_i \left\langle \phi_\mathrm{l}\right|_i - \sum_{k=1}^{g_\mathrm{l}} \left|l_k\right\rangle_i \left\langle l_k\right|_i, \tag{I.24}$$

$$\hat{B}_i' \equiv \sqrt{g_\mathrm{u} g_\mathrm{l}} \left|\phi_\mathrm{u}\right\rangle_i \left\langle \phi_\mathrm{l}\right|_i. \tag{I.25}$$

Here, $\Re\omega$ and $\Im\omega$ in (I.22) are the real and imaginary parts of ω, respectively. By using the representation (I.22), we can obtain the following result:

$$\begin{aligned}\left[\hat{P}_i, \hat{\tau}_i^x\right] &= [\left|\phi_\mathrm{u}\right\rangle_i \left\langle \phi_\mathrm{u}\right|_i, \hat{\tau}_i^x] + [\left|\phi_\mathrm{l}\right\rangle_i \left\langle \phi_\mathrm{l}\right|_i, \hat{\tau}_i^x] \\ &= i\Im\omega(\hat{C}_i^\mathrm{u} + \hat{C}_i^\mathrm{l} + \text{H.c.}),\end{aligned} \tag{I.26}$$

where

$$\hat{C}_i^\mathrm{u} \equiv \frac{1}{c\sqrt{g_u}} \sum_{k=1}^{g_\mathrm{u}} (2k - g_u - 1) \left|\phi_\mathrm{u}\right\rangle_i \left\langle u_k\right|_i,$$

$$\hat{C}_i^\mathrm{l} \equiv \frac{1}{c\sqrt{g_l}} \sum_{k=1}^{g_\mathrm{l}} (2k - g_l - 1) \left|\phi_\mathrm{l}\right\rangle_i \left\langle l_k\right|_i. \tag{I.27}$$

From the result (I.26), we find that (I.17) holds in the case where ω is a real number: $\Im\omega = 0$, which provides

$$\hat{\tau}_i^x = \hat{P}_i \hat{\tau}_i^x \hat{P}_i + (\hat{1}_i - \hat{P}_i)\hat{\tau}_i^x(\hat{1}_i - \hat{P}_i). \tag{I.28}$$

As a result, (I.11) is found to be hold, and the Hamiltonian $\hat{H}$ are reducible and block diagonalizable, independent of the time s as well as a structure of the spin coupling.

Finally, we will prove the lemma II, where the ground state of the initial Hamiltonian $\hat{H}_x$ with $h_i^x > 0$ is an element of the Hilbert space spanned by $\left|\phi_\mathrm{u,l}\right\rangle_i$, if the conditions $\omega \in \mathbb{R}$ and $\omega > -1$ hold. In the following, we assume that the parameter ω is a real number. Since the initial driver Hamiltonian $\hat{H}_x$ is a sum of a single-site spin operator $-h_i^x \hat{\tau}_i^x$ with $h_i^x > 0$, let $\left|\Psi(s=0)\right\rangle \equiv \bigotimes_{i=1}^{N} \left|\psi_0\right\rangle_i$ be the initial ground state, where $\left|\psi_0\right\rangle_i$ is the ground state of $-h_i^x \hat{\tau}_i^x$ with $h_i^x > 0$. In order to prove the lemma II, it is sufficient to show that the state $\left|\psi_0\right\rangle_i$ is an element of the Hilbert space spanned by $\left|\phi_\mathrm{u,l}\right\rangle_i$.

We construct eigenstates of $-\hat{\tau}_i^x$, whose number is $(g_\mathrm{u} + g_\mathrm{l})$, given in the form

$$\left|m_\mathrm{u}\right\rangle_i \equiv \frac{1}{\sqrt{g_\mathrm{u}}} \sum_{k=1}^{g_\mathrm{u}} e^{2\pi i(k-1)m_\mathrm{u}/g_\mathrm{u}} \left|u_k\right\rangle_i \tag{I.29}$$

$$\left|m_\mathrm{l}\right\rangle_i \equiv \frac{1}{\sqrt{g_\mathrm{l}}} \sum_{k=1}^{g_\mathrm{l}} e^{2\pi i(k-1)m_\mathrm{l}/g_\mathrm{l}} \left|l_k\right\rangle_i \tag{I.30}$$

$$\left|\lambda_\pm\right\rangle_i \equiv \sqrt{g_\mathrm{u}} a_\pm \left|\phi_\mathrm{u}\right\rangle_i + \sqrt{g_\mathrm{l}} \left|\phi_\mathrm{l}\right\rangle_i, \tag{I.31}$$

where $m_\mathrm{u,l} = 1, 2, \ldots, g_\mathrm{u,l} - 1$. Eigenvalues of $-\hat{\tau}_i^x$ for $\left|m_\mathrm{u,l}\right\rangle_i$ are given by ω/c. The states $\left|\lambda_\pm\right\rangle_i$ become eigenstates of $-\hat{\tau}_i^x$, if we take

$$a_\pm \equiv \frac{(g_\mathrm{u} - g_\mathrm{l})\omega \mp \sqrt{(g_\mathrm{u} - g_\mathrm{l})^2\omega^2 + 4g_\mathrm{u}g_\mathrm{l}}}{2g_\mathrm{u}}, \tag{I.32}$$

whose eigenvalues of $-\hat{\tau}_i^x$ are given by

$$\lambda_\pm = \frac{1}{c}\left\{\left(1 - \frac{g_\mathrm{u} + g_\mathrm{u}}{2}\right)\omega \pm \frac{1}{2}\sqrt{(g_\mathrm{u} - g_\mathrm{l})^2\omega^2 + 4g_\mathrm{u}g_\mathrm{l}}\right\}. \tag{I.33}$$

These eigenstates $\left|m_\mathrm{u,l}\right\rangle_i$ and $\left|\lambda_\pm\right\rangle_i$ are orthogonal. We can find that the relations $\lambda_+ > \lambda_-$ as well as $(\omega/c) > \lambda_-$ hold in the case where $\omega > -1$. As a result, $h_i^x \lambda_-$ is the minimum eigenvalue of $-h_i^x \hat{\tau}_i^x$ with $h_i^x > 0$, and the ground state is $\left|\lambda_-\right\rangle_i = \sqrt{g_\mathrm{u}} a_- \left|\phi_\mathrm{u}\right\rangle_i + \sqrt{g_\mathrm{l}} \left|\phi_\mathrm{l}\right\rangle_i$, which indicates that the initial ground state is an element of the Hilbert space spanned by $\left|\phi_\mathrm{u,l}\right\rangle_i$.

To summarize, in the case where ω is a real number and $\omega > -1$ holds, the quantum annealing problem in the quantum Wajnflasz–Pick model can be exactly described by the reduced Hilbert space spanned by $|\phi_{\mathrm{u,l}}\rangle_i$.

As in panel (b) in Fig. 5, we can find that the level crossing with respect to the ground state emerges twice while annealing. It might be expected as the emergence of the double first order phase transition while annealing discussed in Ref. [1], where the system may come back to the ground state at the end of the annealing. However, there is no Landau–Zener tunneling between them, because we find no matrix elements between these level crossing states. In the case where $\Im\omega \neq 0$, non-zero value of matrix elements emerges between the Hilbert space projected by $\hat{P}$ and their orthogonal complement, which provides the anti-crossing of energy levels. In this case, discussion of the quantum annealing will become more complicated than that in the present study. At non-zero temperature case, the Schrödinger dynamics employed in this paper is not applicable, and it will be important to consider lower energy states in the Hilbert space projected by $\hat{P}$ as well as those in their orthogonal complement.

[1] Seki, Y., Tanaka, S. & Kawabata, S., Quantum Phase Transition in Fully Connected Quantum Wajnflasz–Pick Model. *J. Phys. Soc. Jpn.* **88**, 054006 (2019).
